# Supplementary material for: Theranostic Agent Combining Fullerene Nanocrystals and Gold Nanoparticles for Photoacoustic Imaging and Photothermal Therapy
Source: Int J Mol Sci. 2022 Apr 23;23(9):4686. doi: 10.3390/ijms23094686 (PMC9105889; doi:10.3390/ijms23094686)
Supplement: Supplementary file 1 [file ijms-23-04686-s001.zip › ijms-1703351-supplementary.pdf]

Supporting information

Theranostic Agent Combining Fullerene Nanocrystals and Gold Nanoparticles for Photoacoustic Imaging and Photothermal Therapy

Riku Kawasaki,[a,*,‡] *Kosuke Kondo,*[a,‡] *Risako Miura,*[b,‡,*] *Keita Yamana,*[a] *Hinata Isozaki,*[a] *Risako Shimada,*[a] *Shogo Kawamura,*[a] *Hidetoshi Hirano,*[a] *Tomoki Nishimura,*[d] *Naoki Tarutani,*[a] *Kiyofumi Katagiri,*[a] *Alexandra Stubelius,*[c] *Shin-ichi Sawada,*[d] *Yoshihiro Sasaki,*[d] *Kazunari Akiyoshi,*[d] *and Atsushi Ikeda*[a,*]

a) Applied Chemistry Program, Graduate School of Advanced Science and Engineering, Hiroshima University, 1-4-1 Kagamiyama, Higashi-Hiroshima, 739-8527, Japan.

b) Department of Energy and Hydrocarbon Chemistry, Graduate School of Engineering, Kyoto University, Kyoto Daigaku Katsura, Nishikyo-ku, Kyoto City, 615-8510, Japan.

c) Division of Chemical Biology, Department of Biology and Biological Engineering, Chalmers University of Technology, SE-412 96 Gothenburg, Sweden.

d) Department of Polymer Chemistry, Graduate School of Engineering, Kyoto University, Kyoto Daigaku Katsura, Nishikyo-ku, Kyoto City, 615-8510, Japan. d) Department of Chemistry and Materials, Faculty of Textile Science and Technology, Shinshu University, 3-15-1 Tokida, Ueda, 386-8567, Japan.

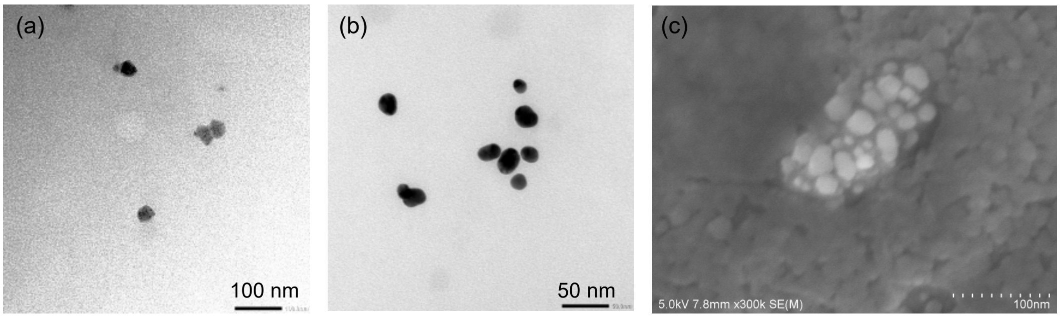

Figure S1 Representative morphology of FNCs (a) and GNPs (b). The samples were observed without staining by TEM (accelerate voltage, 80 kV). (c) Representative morphology of FGNPs which was observed by FE-SEM (accelerate voltage, 80 kV).

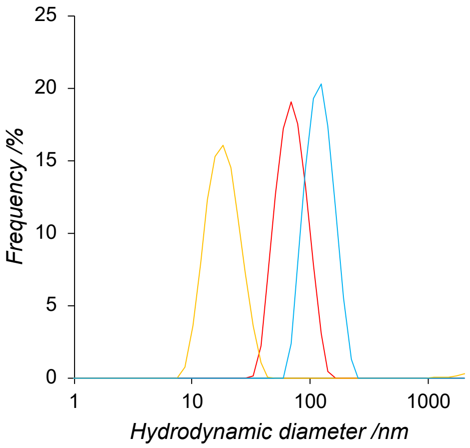

Figure S2 Size distribution of FNCs, GNPs, and FGNPs. The FNCs (red), GNPs (yellow), and FGNPs (blue) were dispersed in MilliQ (pH, 7.4; 25 ºC).

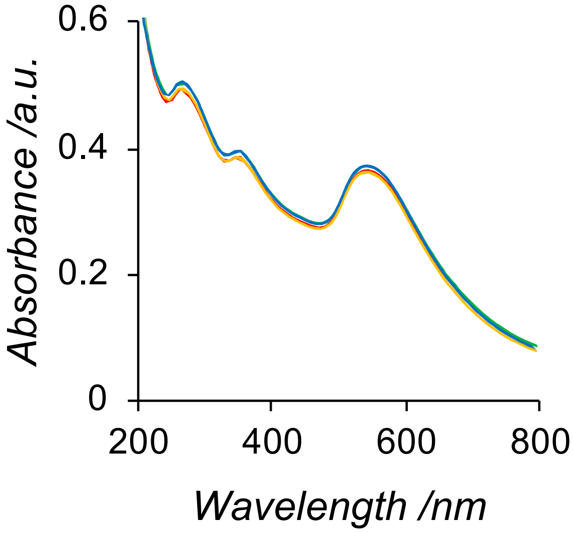

Figure S3 Stability of FGNPs in aqueous media. The absorption spectra changes were measured by UV-Vis spectrometer. (Red, 0 d; Yellow, 1 d; Green, 3 d; Blue, 7 d).

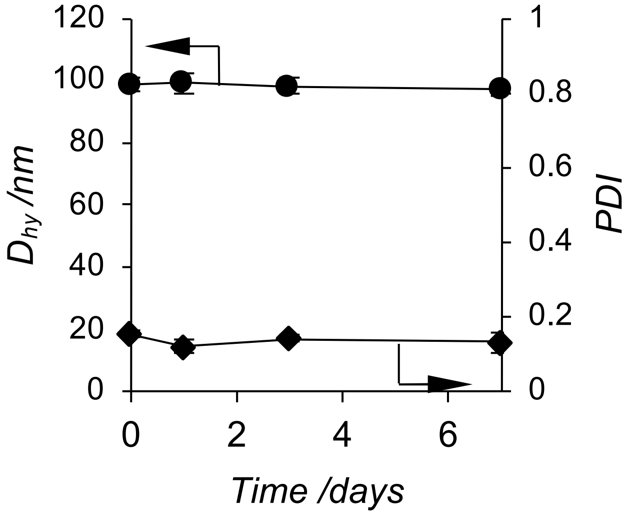

Figure S4 Colloidal stability of FGNPs in aqueous media. Changes in hydrodynamic diameter (Circle symbols) of FGNPs and PDI value (Diamond Symbols) were measured by DLS. Data represents mean ± SD. The samples were prepared in MilliQ (pH, 7.4, 25 °C)

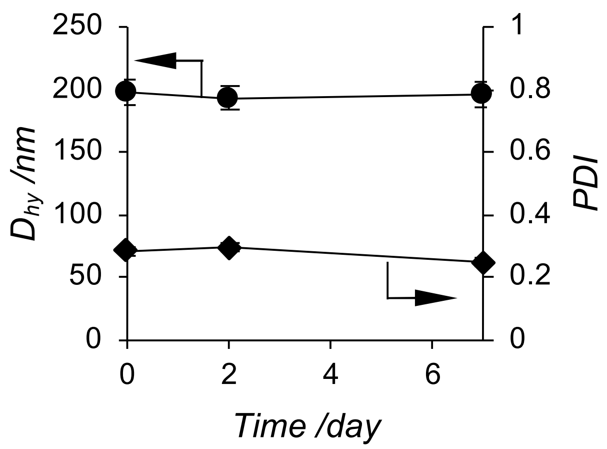

Figure S5 Colloidal stability of FGNPs in culture media containing serum protein. Changes in hydrodynamic diameter (Circle symbols) of FGNPs and PDI value (Diamond Symbols) were measured by DLS. Data represents mean ± SD.

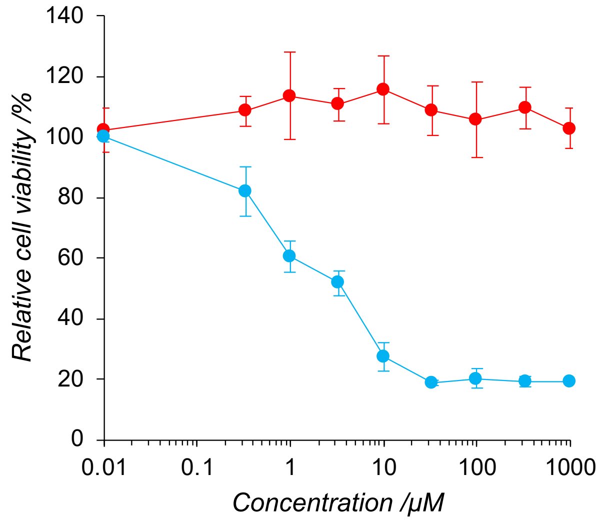

Figure S6 Photo-induced cytotoxicity toward Colon26 cells using FNCs (red) and FGNPs (blue). The cells were co-incubated with FNCs or FGNPs with varying concentration for 24 h. The cells were exposed to light with optimal wavelength (>620 nm) for 30 mins. After 24 h incubation, the cell viability was confirmed by Cell Counting Kit-8 (n=3). Data represents mean ± SD.
